# Genomic alterations and clinical outcomes in patients with dedifferentiated liposarcoma

**DOI:** 10.1002/cam4.5502

**Published:** 2022-12-04

**Authors:** Megan H. Jagosky, Colin J. Anderson, James T. Symanowski, Nury M. Steuerwald, Carol J. Farhangfar, Emily A. Baldrige, Jennifer H. Benbow, Michael B. Livingston, Joshua C. Patt, Will A. Ahrens, Jeffrey S. Kneisl, Edward S. Kim

**Affiliations:** ^1^ Department of Solid Tumor Oncology, Levine Cancer Institute, Carolinas Medical Center, Atrium Health Charlotte North Carolina USA; ^2^ Department of Orthopedic Oncology, Musculoskeletal Institute, Atrium Health Charlotte North Carolina USA; ^3^ Department of Biostatistics, Levine Cancer Institute, Carolinas Medical Center Atrium Health Charlotte North Carolina USA; ^4^ The Molecular Biology and Genomics Laboratory, Levine Cancer Institute, Carolinas Medical Center, Atrium Health Charlotte North Carolina USA; ^5^ LCI Translational Research, Levine Cancer Institute, Carolinas Medical Center, Atrium Health Charlotte North Carolina USA; ^6^ LCI Research Support, Clinical Trials Office, Levine Cancer Institute, Carolinas Medical Center, Atrium Health Charlotte North Carolina USA; ^7^ City of Hope Comprehensive Cancer Center Duarte California USA; ^8^ Department of Pathology Levine Cancer Institute, Carolinas Medical Center, Atrium Health Charlotte North Carolina USA

**Keywords:** liposarcoma, next‐generation sequencing, NGS, precision oncology, targeted therapy

## Abstract

**Purpose:**

Patients with unresectable dedifferentiated liposarcoma (DDLPS) have poor overall outcomes. Few genomic alterations have been identified with limited therapeutic options.

**Experimental Design:**

Patients treated at Levine Cancer Institute with DDLPS were identified. Next generation sequencing (NGS), immunohistochemistry (IHC), and fluorescence in situ hybridization (FISH) testing were performed on tumor tissue collected at diagnosis or recurrence/progression. Confirmation of genomic alterations was performed by orthologous methods and correlated with clinical outcomes. Univariate Cox regression was used to identify genomic alterations associated with clinical outcomes.

**Results:**

Thirty‐eight DDLPS patients with adequate tissue for genomic profiling and clinical data were identified. Patient characteristics included: median age at diagnosis (66 years), race (84.2% Caucasian), and median follow‐up time for the entire cohort was 12.1 years with a range from approximately 3.5 months to 14.1 years. Genes involved in cell cycle regulation, including MDM2 (74%) CDK4 (65%), and CDKN2A (23%), were amplified along with WNT/Notch pathway markers: HMGA2, LGR5, MCL1, and CALR (19%–29%). While common gene mutations were identified, PDE4DIP and FOXO3 were also mutated in 47% and 34% of patients, respectively, neither of which have been previously reported. FOXO3 was associated with improved overall survival (OS) (HR 0.37; *p* = 0.043) along with MAML2 (HR 0.30; *p* = 0.040). Mutations that portended worse prognosis included RECQL4 (disease‐specific survival HR 4.67; *p* = 0.007), MN1 (OS HR = 3.38; *p* = 0.013), NOTCH1 (OS HR 2.28, *p* = 0.086), and CNTRL (OS HR 2.42; *p* = 0.090).

**Conclusions:**

This is one of the largest retrospective reports analyzing genomic aberrations in relation to clinical outcomes for patients with DDLPS. Our results suggest therapies targeting abnormalities should be explored and confirmation of prognostic markers is needed. Dedifferentiated liposarcoma is one of the most common subtypes of soft tissue sarcoma yet little is known of its molecular aberrations and possible impact on outcomes. The work presented here is an evaluation of genetic abnormalities among a population of patients with dedifferentiated liposarcoma and how they corresponded with survival and risk of metastases. There were notable gene mutations and amplifications commonly found, some of which had interesting prognostic implications.

## 1 INTRODUCTION

Soft tissue sarcomas (STS), neoplasms derived from mesenchymal cells, have traditionally been challenging to study and treat due to their rarity and diversity. STS make up 1% of all cancers and have over 50 histologic subtypes.^1^ Liposarcoma (LPS), the most common STS subtype, comprises approximately 20% of all STS cases and is subcategorized based on histologic and pathologic features into myxoid/round cell, pleomorphic, well‐differentiated (WDLPS), and dedifferentiated LPS (DDLPS). The most common of these subtypes are WDLPS and DDLPS which comprise 48%–58% of all LPS.^1^

DDLPS, occurring most often in the retroperitoneal space, can be a challenge to treat due to its aggressive, high‐grade nature with three‐year local and metastatic recurrence rates of 80% and 30%, respectively.^2^ Complete resection is often difficult in retroperitoneal DDLPS as tumors often surround essential structures.^3^ For patients with metastatic DDLPS, standard of care first‐line therapy is doxorubicin with a modest response rate of 11%.^4^ While the addition of other cytotoxic agents, like ifosfamide, have shown improvements in response rates and progression‐free survival in multiple studies, they are often associated with increased toxicity and lack of significant improvements in survival outcomes.^5^, ^6^ Second‐line systemic therapies have led to modest improvements in outcomes with trabectedin providing a median 3 month longer time to progression compared to dacarbazine with similar overall survival. A study of later‐line eribulin mesylate showed that in the liposarcoma subgroup, PFS was approximately 47% at 12 weeks but only 2 of the 32 patients had a response.^7^, ^8^ Readily accessible tumor genetic sequencing has led to development of targeted therapies and expanded treatment options, including revolutionary drugs targeting KIT proto‐oncogene and platelet‐derived growth factor receptor A (PDGFRa) aberrations in gastrointestinal stromal tumors and dermatofibrosarcoma protuberans.^9^

Genetic sequencing of DDLPS has led to the discovery of a unique highly amplified supernumerary ring/giant cell marker chromosome 12q14‐15, found in approximately 90% of all cases.^10^ Genes on this ring include mouse double minute 2 homolog (MDM2) (12q15), high mobility group AT‐hook 2 (HMGA2) (12q14.3) and cyclin dependent kinase 4 (CDK4) (12q14.1).^10^ The presence of MDM2 amplification distinguishes WDLPS/DDLPS from benign lipomas. Additionally, the presence of MDM2 with CDK4 amplification provides both a sensitive (97% and 92%, respectively), and specific (83% and 95%, respectively), marker for diagnosis of WDLPS/DDLPS.^11^ Incidence reported by the Memorial Sloan Kettering Cancer Center (MSKCC) cBioPortal,^12^, ^13^ for CDK4, HMGA2, and MDM2 amplification is 70%, 60%, and 75%, respectively, among DDLPS patients. DDLPS may have additional genetic aberrations that lead to its more aggressive phenotype.

While gene alterations and amplifications in LPS have been described, there is a paucity of data from real world specimens. Additionally, limited information regarding prognostic implications of genetic abnormalities is available. We conducted one of the largest single institution retrospective analyses including demographic and clinical outcomes data from patients diagnosed with retroperitoneal DDLPS. This study assessed genomic profiling of tumors from DDLPS patients in conjunction with RNA expression profiling to identify potential prognostic biomarkers.

## 2 METHODS

This retrospective, single‐institution study examined patients with pathologically confirmed retroperitoneal DDLPS diagnosed at Levine Cancer Institute between 1996 and 2016. Inclusion criteria included patients 18 years or older with complete DDLPS medical histories. Patient demographics and clinical characteristics including sex, age, race, smoking history, and disease‐specific data were acquired from patients' electronic medical record. This study was approved by the Institutional Review Board at Atrium Health (LCI‐RARE‐MP‐001R, approved October 6th, 2015). Due to the retrospective nature of this study and minimal risk to patients, the informed consent was waived.

LPS tissue collected during standard of care biopsies and stored in the Levine Cancer Institute Biospecimen Repository were prepared as formalin‐fixed paraffin‐embedded tissue on unstained slides. The molecular findings for this study were obtained using results from testing done for treatment purposes during routine patient care; for samples where the molecular testing was not available, the results were acquired by sending banked samples for analysis at no expense to the patient. The analysis was paid for by using funds received for the study by the Paula Takacs Foundation. A majority of tumor specimens had both well‐differentiated and dedifferentiated components, and the reviewing expert sarcoma pathologist isolated the higher‐grade dedifferentiated section and specifically sent for the molecular testing. All samples sent by the pathologist had MDM2 FISH amplification confirmed. A total of forty‐eight specimens were sent using standard lab protocols to a Clinical Laboratory Improvement Amendments‐certified laboratory (Caris Life Sciences) for genomic profiling of 592 genes via Caris COE Mi Profile X platform. All personnel at Caris Life Sciences were blinded to any clinical data associated with these samples. Immunohistochemistry (IHC) analysis was performed on the following genes of interest: anaplastic lymphoma kinase (ALK), androgen receptor (AR), tyrosine protein kinase Met (cMET), epidermal growth factor receptor (EGFR), estrogen receptor (ER), excision repair cross complementation group 1 (ERCC1), human epidermal growth factor receptor (HER2), methylguanine‐DNA methyl‐transferase (MGMT), mutL homolog 1 (MLH1), mutS homolog 2 (MSH2), mutS homolog 6 (MSH6), programmed cell‐death protein 1 (PD‐1), programmed death‐ligand 1 (PD‐L1), progesterone receptor (PR), phosphatase and tensin homolog (PTEN), ribonucleotide reductase catalytic subunit 1 (RRM1), transducing‐like enhancer protein 3 (TLE3), DNA topoisomerase 2‐alpha (TOP2A), topoisomerase 1 (TOPO1), tumor suppressor (TS), and tubulin beta 3 class III (TUBB3).

Orthogonal testing using polymerase chain reaction (PCR) and Sanger sequencing were performed to confirm the presence of mutations at the chromosomal locations identified by the Caris panel during next generation sequencing (NGS). Clinical outcomes, including time to local recurrence, time to metastatic disease, recurrence‐free survival (RFS), disease‐specific survival (DSS), and overall survival (OS), were evaluated to identify potential prognostic genomic aberrations. These time‐to‐event endpoints were calculated from the diagnosis date until the date of the event of interest or censored at the date of last disease assessment or last contact. Genes included in final statistical analyses were limited to those that had at least four subjects with the genomic aberration and four subjects without the genomic aberration. Kaplan–Meier techniques were utilized to estimate survival probabilities over time and comparisons between aberration cohorts were evaluated with log‐rank test. Cox Proportional‐Hazards models were used estimate hazard ratios (HR) between aberration cohorts. Due to the exploratory nature of this study and limited sample size, multivariable model selection was not performed. Potential prognostic genes were identified based on a 2‐sided alpha = 0.10 significance level, with no adjustment for multiplicity.

## 3 RESULTS

Forty‐eight (48) subjects diagnosed with DDLPS treated at our institution with tumor specimens available for NGS were identified. Of these, 38 had sufficient tumor tissue for third party profiling and complete clinical data were available. Adequate tumor sample was available to perform copy number variation (CNV) testing, gene mutational analysis, and IHC analyses on 31, 32, and 38 subject specimens, respectively.

### 3.1 Common molecular aberrations

Evaluation of CNV revealed twelve commonly over‐amplified genes (present in four or more specimens). Disease‐related gene variations known to be present on the 12q amplicon, 12q14‐15, were amplified among our population, including MDM2 (74%), CDK4 (65%), and HMGA2 (29%). WNT/Notch pathway markers including leucine‐rich repeat‐containing G protein‐coupled receptor 5 (LGR5) (23%), induced myeloid leukemia cell differentiation protein (MCL1) (23%), and cyclin‐dependent kinase inhibitor 2A (CDKN2A) (23%) were also amplified. Additionally, calreticulin (CALR) was over‐expressed in 19% of tumor samples.

Among the 592 genes tested using NGS, 25 were commonly (present in four or more specimens) mutated in the 32 DDLPS patient tumor samples. The most mutated genes include phosphodiesterase 4D Interacting Protein (PDE4DIP; 47%), Forkhead box class O (FOXO3; 34%), LDL Receptor Related Protein 1B (LRP1B; 31%), RecQ Like Helicase 4 (RECQL4; 25%), Mastermind Like Transcriptional Coactivator 2 MAML2 (22%), and centriolin (CNTRL; 22%), NOTCH1/2 (19%), and Pericentriolar Material 1 (PCM1; 19%), among others.

Of the 21 genes evaluated for expression with IHC, all genes were expressed in at least one specimen except for ALK and HER2. The top five genes with the highest expression patterns identified via IHC analysis included PTEN (79%), TS (63%), TUBB3 (58%), PD1 (50%), and TOP2A (47%).

### 3.2 Outcomes

The clinical cohort (*N* = 38) included 27 male subjects (71.1%) with a median age at diagnosis of 66 years (range: 30–85) and Caucasians comprising 84.2%. Basic demographic and disease‐related factors are summarized in Table 1. Median follow‐up times for CNV, mutational status, and IHC cohorts were 12.1 years (3.5 months—14.1 years), 11.4 years (3.5 months—14.1 years), and 12.1 years (3.5 months—14.1 years), respectively. OS and time to distant recurrence were found to be significantly associated with genetic alterations.

**TABLE 1:** Subject demographic and disease characteristics

	*N* = 38
Age (years), Median [Range]	66 (30–85)
Sex, no. (%)	
Male	27 (71.1%)
Female	11 (28.9%)
Race, *n* (%)	
Caucasian	32 (84.2%)
African American	3 (7.9%)
Other/Unknown	3 (7.9%)
Smoking history, *n* (%)	
Never smoker	14 (36.8%)
Current smoker	5 (13.2%)
Former smoker	14 (36.8%)
Unknown	5 (13.2%)
Pathologic stage, *n* (%)	
II	2 (5.3%)
III	31 (81.6%)
IV	5 (13.2%)
Histologic grade, *n* (%)	
II	14 (36.8%)
III	24 (63.2%)
Tumor size, *n* (%)	
<5 cm	3 (7.9%)
≥5, <10 cm	12 (31.6%)
≥10, <15 cm	7 (18.4%)
≥15 cm	16 (42.1%)
Primary tumor site, *n* (%)	
Abdomen	2 (5.3%)
Arm	2 (5.3%)
Retroperitoneum	22 (57.9%)
Pelvis	5 (13.2%)
Thigh	3 (7.9%)
Other	4 (10.5%)

**FIGURE 1:**
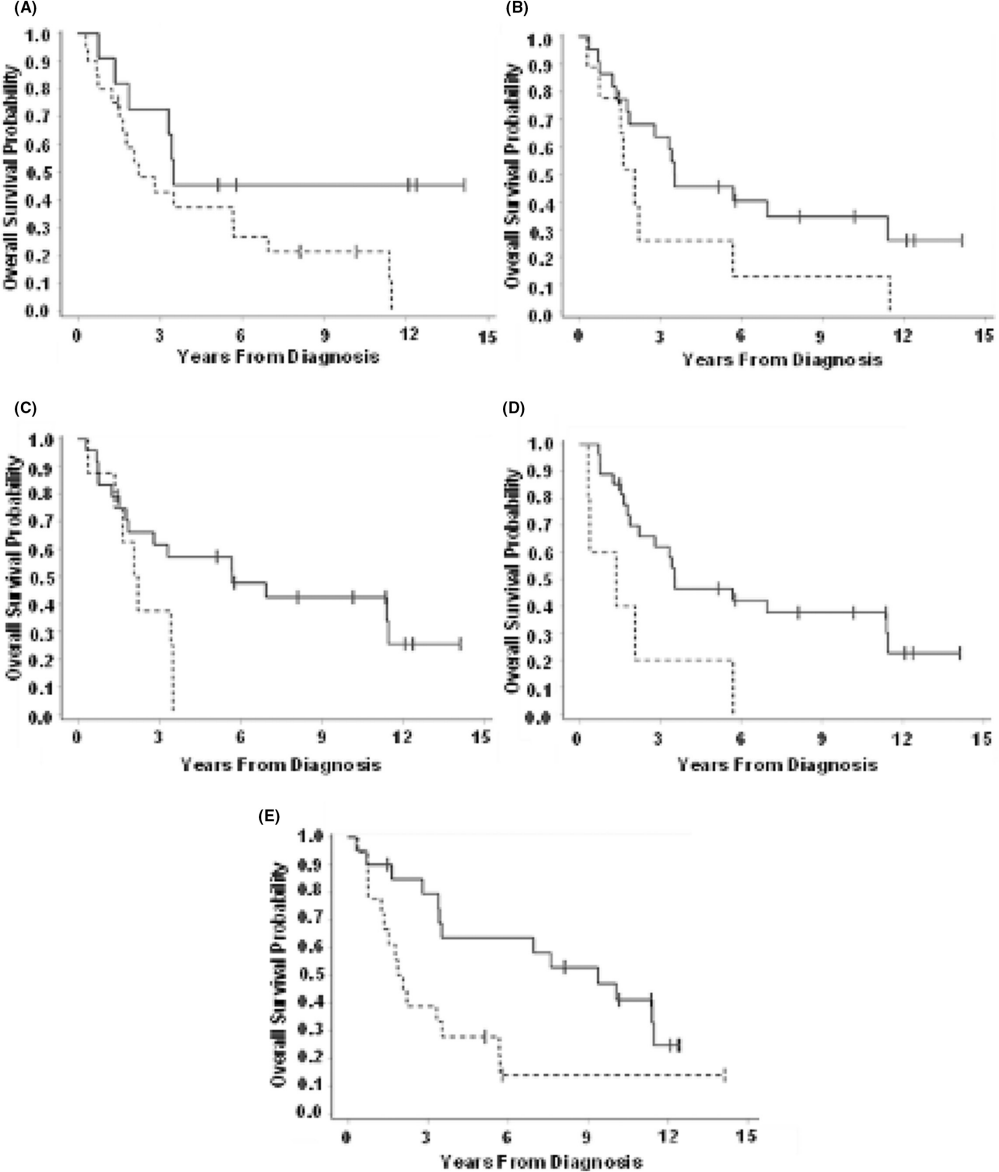
Biomarkers associated with decreased overall survival (A) CDK4 (HR 2.22; *p* = 0.090), (B) HMGA2 (HR 2.11; *p* = 0.086), (C) REQL4 (HR 2.93; *p* = 0.019), (D) MN1 (HR 3.38; *p* = 0.013), and (E) TOP2A (HR 2.65; *p* = 0.012).

The amplification of CDK4 (*p* = 0.090) and MDM2 (*p* = 0.067) was found to be associated with an increased risk of metastatic disease (Table 2). CDK4 was also associated with decreased OS (HR 2.22; *p* = 0.090) (Figure 1). Additionally, the amplification of HMGA2 was associated with an increased risk for metastasis (HR 4.18; *p* = 0.058) and decreased OS (HR 2.11; *p* = 0.086).

**TABLE 2:** Copy number variation (*N* = 31)

Gene (Incidence)	HR	95% Confidence Interval	*p*‐value
Time to metastatic disease (Number of events = 6)			
MDM2 (23)	NE	NE	0.067
CDK4 (20)	NE	NE	0.080
HMGA2 (9)	4.18	0.84–20.89	0.058
Overall survival (Number of events = 23)			
CDK4 (20)	2.22	0.86–5.73	0.090
HMGA2 (9)	2.11	0.88–5.06	0.086

Several gene mutations identified were associated with multiple disease‐specific adverse outcomes (Table 3). RECQL4 mutations were associated with worse OS (HR 2.93; *p* = 0.019) and DSS (HR 4.67; *p* = 0.007), in addition to the decreased time to local recurrence (HR 7.04; *p* = 0.003) and time to metastatic recurrence (HR 12.55; *p* = 0.015) (Figure 1). MN1 mutations were associated with reduced time to local recurrence of (HR 23.50; *p* = 0.001), RFS (HR 4.68; *p* = 0.032), and OS (HR 3.38; *p* = 0.013). Additionally, NOTCH1 mutations were associated with decreased time to local recurrence (HR 3.84; *p* = 0.051) and OS (HR 2.28; *p* = 0.086). Lastly, the risk of death was increased by mutations in CNTRL (HR 2.42; *p* = 0.090).

**TABLE 3:** Gene mutations (*N* = 32)

Gene (Incidence)	HR	95% Confidence Interval	*p*‐value
Time to local recurrence (Number of events = 11)			
FOXO3 (11)	0.28	0.06–1.32	0.087
RECQL4 (8)	7.04	1.62–30.66	0.003
NOTCH1 (6)	3.84	0.90–16.38	0.051
MN1 (5)	23.50	1.47–375.68	0.001
Time to metastatic disease (Number of events = 6)			
RECQL4 (8)	12.55	1.03–152.77	0.015
Recurrence‐free survival (Number of events = 15)			
RECQL4 (8)	2.80	1.20–12.05	0.015
MN1 (5)	4.68	0.99–22.19	0.032
KAT6A (4)	NE	NE	0.076
Disease‐specific survival (Number of events = 14)			
RECQL4 (8)	4.67	1.39–15.73	0.007
MAML2 (7)	0.16	0.02–1.25	0.047
Overall survival (Number of events = 23)			
FOXO3 (11)	0.37	0.18–1.01	0.043
RECQL4 (8)	2.93	1.14–7.52	0.019
MAML2 (7)	0.30	0.09–1.01	0.040
CNTRL (7)	2.42	0.84–6.96	0.090
NOTCH1 (6)	2.28	0.87–5.97	0.086
MN1 (5)	3.38	1.22–9.38	0.013

**FIGURE 2:**
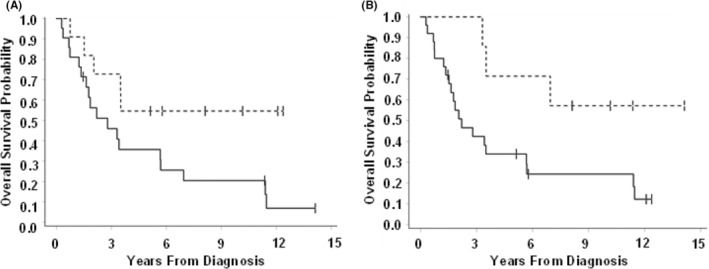
Biomarkers associated with increased overall survival (A) FOXO3 (HR 0.37; *p* = 0.043) and (B) MAML2 (HR 0.30; *p* = 0.040).

Our analysis also identified favorable gene mutations (Figure 2). Mutations in FOXO3 lead to increased time to local recurrence (HR 0.28; *p* = 0.087) and improved OS (HR 0.37; *p* = 0.043). The presence of mutations in the MAML2 gene was associated with improved DSS (HR 0.16; *p* = 0.047) and OS (HR 0.30; *p* = 0.040).

Lastly, three genes over‐expressed on IHC were found to be prognostic for worse clinical outcomes (Table 4). TOP2A was associated with a decreased time to metastatic disease (HR 4.13; *p* = 0.044), decreased DSS (HR 3.51; *p* = 0.014), and decreased OS (HR 2.65; *p* = 0.012). TS expression was associated with decreased DSS (HR 2.98; *p* = 0.050) and OS (HR 2.08; *p* = 0.067). TOPO1 expression was associated with worse OS (HR 2.09; *p* = 0.092).

**TABLE 4:** Immunohistochemistry mutations (*N* = 38)

Gene (Incidence)	HR	95% Confidence interval	*p*‐value
Time to metastatic disease (Number of events = 8)			
TOP2A (18)	4.13	0.94–18.11	0.044
Disease‐specific survival (Number of events = 16)			
TS (24)	2.98	0.95–9.35	0.050
TOP2A (18)	3.51	1.23–10.07	0.014
Overall survival (Number of events = 28)			
TS (24)	2.08	0.94–4.64	0.067
TOPA2A (18)	2.65	1.21–5.82	0.012
TOPO1 (8)	2.09	0.87–5.02	0.092

## 4 DISCUSSION

To the best of our knowledge, this is one of the largest retrospective analysis evaluating genetic alterations and their impact on prognosis in DDLPS. While our results are in line with current literature, we did identify some unique and intriguing findings that warrant confirmation from additional datasets. Genetic changes, including large scale chromosome CNVs and smaller scale single nucleotide variants (SNV), were noted among our patients.

Several genes involved in cell cycle regulation were amplified or mutated including MDM2, CDK4, and CDKN2A/p16/INK4A.^14^ As expected, the most amplified genes (MDM2, CDK4, HMGA2) were those present in the chromosome12q13–15 region which is indicative of DDLPS diagnosis. MDM2 and CDK4 play critical roles in the cell cycle.^15^ MDM2, known to promote tumorigenesis via inhibition of the tumor suppressor gene TP53, was the most amplified gene (73%) identified in DDLPS tumor samples. While amplified MDM2 was identified in all patients with metastasis, it was not definitively associated with survival outcomes. The cyclin dependent kinase, CDK4, was also found to be associated with metastatic disease recurrence in our patient population. Hyperactivation of CDK4 leads to progression of the cell cycle through G1 into S phase and initiates unbridled cellular proliferation.^15^ Consistent with what is reported in the literature, CDK4 amplification appeared to impact survival in our patient population.^16^ Specifically, patients with this abnormality were at twice the risk of death compared to those without the mutation (OS HR 2.22). CDK4 and its relative CDK6 are regulated by CDKN2A encoded proteins p14(ARF) and p16(INK4A). Counter‐intuitively, amplification of the CDKN2A^17^ locus was also identified in DDLPS tumors (23%). The amplification of CDKN2A and CDK4 was not mutually exclusive events in our analysis, with some tumors having increased CNV in both domains.

The WNT/Notch pathway has also been implicated in tumorigenesis leading to perpetual stem cell proliferation and self‐renewal.^18^ While numerous genes involved in this pathway were either amplified or mutated in our patient population (HMGA2, LGR5, MCL1, CALR, NOTCH1), HMGA2 was the only gene whose amplification portended a worse prognosis. HMGA2 is a non‐histone architectural mesenchymal transcription factor that is involved in mesenchymal differentiation and proliferation. Its aberrant expression is noted in both benign mesenchymal tumors and malignant tumors. It interestingly has been described in epithelial tumors representing a potential induction of epithelial to mesenchymal transition in which the cells are able to progress and metastasize.^19^ HMGA amplification demonstrated decreased OS (*p* = 0.086) and increased risk of metastatic disease (*p* = 0.058). Like HMGA2, mutations in NOTCH1 also portended worse prognosis with decreased OS (*p* = 0.086) and decreased time to local recurrence (*p* = 0.051).

Numerous gene mutations identified in this study had prognostic implications. First, RECQL4 stands out as a particularly problematic gene mutation, occurring in 25% of our patient population, portended worse OS, DSS, and decreased time to local recurrence and metastatic recurrence. RECQL4 encodes a crucial DNA helicase known to unwind DNA during the replication process.^20^ Its activity is necessary for numerous intracellular regulatory pathways including initiation of DNA replication, maintenance of genomic stability, and transcription. Alteration in RECQL4 has been reported in numerous cancers and disorders including adenocarcinoma of prostate, breast, and colon, with worsened prognosis in gastric cancer^21^, ^22^

Mutations in MN1 also demonstrated poor prognostic outcomes including significantly increased risk of local recurrence (HR 23.5; *p* = 0.001) which translated into significantly decreased RFS (HR 4.5, *p* = 0.032) and OS (HR 3.38; *p* = 0.013). The oncogenic function of MN1 has not been fully expounded but it is a known transcription co‐activator, interacting with retinoic acid receptor, RAR‐RXR. While mutations in MN1 have previously been implicated in acute myeloid leukemia (AML) and meningioma,^23^, ^24^ it has not been reported in STS. CNTRL, another gene with little known about its role in malignancy, was identified in 22% of DDLPS tumors and portended a worse OS (*p* = 0.090). CNTRL is needed for centrosome function as a microtubule organizer^25^ and its translocation with fibroblast growth factor receptor 1 has been described in a myeloproliferative disorder.^26^

While most genetic mutations portended worse prognosis, FOXO3 mutation (34%) was associated with significant improvement in OS (HR 0.37; *p* = 0.043). FOXO3 is a transcription factor, involved in both transcription activation and repression, dictating cell fate. It is regulated by multiple pathways including PI3K‐PKB, Ras–Raf–MEK–ERK, IKK, and AMPK.^27^ In the presence of growth factors, FOXO protein is degraded but in the absence of growth factors FOXO protein is translocated to the nucleus where it up regulates genes to promote cell cycle arrest and cell death. Mutations in FOXO genes are involved in chromosomal translocations leading to proto‐oncogenic fusions in AML and rhabdomyosarcoma. Somatic mutations have also been identified in lymphoid malignancies.^28^ Over‐expression of FOXO is associated with poor prognosis in glioblastoma, gastric cancer, hepatocellular cancer, and triple negative breast cancer whereas under‐expression is associated with poor prognosis in glioma and ovarian cancer.^29^, ^30^, ^31^ In our DDLPS patient population, FOXO3 mutation was associated with improved outcomes.

In addition to FOXO3, mutations in MAML2 (22%) were also commonly found and demonstrated improved prognosis. It portended increased OS (HR 0.30; *p* = 0.04) and DSS (HR 0.16; *p* = 0.047). MAML2 encodes a protein belonging to a family of transcriptional activators leading to coactivation of all four Notch receptors important for activation of target genes. MAML2 can be highly expressed in B cell‐derived lymphomas, mucoepidermoid carcinomas, and chronic lymphocytic leukemia.^32^

Our analysis in conjunction with current literature highlights the importance of genetic sequencing in prognostication and identification of potentially targetable mutations in STS patients.^33^, ^34^, ^35^ A recent report by Boddu et al. found that of 114 STS cases, 49.1% had actionable mutations. On average, three driver mutations per tumor were identified with the most common gene alterations including TP53 (36.8%), CDKN2A/B (20.2%), CDK4/MDM2 (19.3%), ATRX (13.2%), and RB1 (13.2%).^33^ A review of 102 advanced STS patients with molecular profiling demonstrated all DDLPS and WDLPS tumors had actionable mutations, unsurprisingly again the most common were MDM2 and CDK4.^34^ These studies are similar to ours wherein 15.6% of our patients had gene alterations in TP53 and ATRX and 22.6% had gene alterations in CDKN2A/B. MDM2 and CDK4 genes had the greatest incidence of alterations at 74.2% and 64.5%, respectively.

Drugs that target commonly amplified genes in DDLPS, including MDM2 and CDK4, are currently available or in development. The clinical efficacy of MDM2 inhibition has been modest in STS and other tumor subtypes. A phase 1 trial presented at the 2018 annual American Society of Clinical Oncology meeting used an oral MDM2 inhibitor, DS‐3032b to treat LPS, lymphoma, and solid tumors. While the drug was tolerable there was only one LPS patient with partial response and stable disease as best response was seen in 60% of the 79 evaluable patients.^36^ Median duration of stability was 6.7 months (range: 1.6 to 36.4 months). Other MDM2 inhibitors are in the pipeline and preclinical models have shown synergism when combining MDM2 inhibition with CDK4/6 inhibitors, leading to the potential for future clinical trials.^37^, ^38^

CDK4/6 inhibitors have demonstrated favorable outcomes in the treatment of LPS. The CDK4/6 inhibitor Palbociclib has been studied in the treatment of WDLPS and DDLPS demonstrating median PFS of 17.9 weeks at the 125 mg daily dose (21 days of 28‐day cycle). It is listed as category 2A for use in this group of patients. Abemaciclib, a stronger CDK4 inhibitor, was studied in 24 patients with DDLPS in a recent phase II trial (NCT02846987). Patients received it as either first‐line or later. PFS at 12 weeks was 74% with a median PFS of 30 weeks. One PR was achieved. A larger, phase III randomized trial (abemaciclib vs. placebo) is currently underway (SARC041).^39^ Numerous other early‐stage trials are ongoing using another CDK4/6 inhibitor ribociclib, pairing it with the protein kinase inhibitor everolimus in DDLPS and leiomyosarcoma,^40^ and ribociclib and doxorubicin in metastatic/advanced STS.^40^, ^41^

## 5 CONCLUSION

This genomic profiling in retroperitoneal DDLPS. By limiting the analysis to one disease location and including only dedifferentiated tissue, selected out by a sarcoma‐specialized pathologist, we avoided confounding results including factors related to extremity LPS' association with better prognosis, easier resection, and less risk of margin positivity.^42^ While thorough quality assurance was utilized, limitations of this study include biases inherent to retrospective analyses. Additionally, some of the samples were unable to have all molecular testing obtained due to limitations on quantity of specimen.

Commonalities uncovered among DDLPS specimens included alterations in genes involved in the cell cycle (MDM2, CDK4, CDKN2A) of which CDK4 amplification demonstrated significantly decreased survival. Gene aberrations that impact the Wnt/NOTCH pathway were also commonly identified in our patient cohort. Some of these alterations portended worse prognosis (HMGA2, NOTCH1) while others were protective (MAML2). Lastly, other common gene mutations of interest included RECQL4, MN1, and CNTRL1 that portended worse prognosis and may be helpful in clinical decision making related to how aggressive to be in treatment planning. Large scale molecular analyses on individual sarcoma subtypes are necessary given the vast heterogeneity in histology and behavior. These analyses provide better understanding of disease biology and can uncover prognostic and targetable genetic alterations as ours did.

## AUTHOR CONTRIBUTIONS

**Megan Jagosky:** Data curation (lead); formal analysis (supporting); investigation (lead); supervision (equal); writing – original draft (equal); writing – review and editing (equal). **Colin J. Anderson:** Data curation (equal); investigation (equal); writing – original draft (supporting). **James T Symanowski:** Formal analysis (lead); methodology (equal); writing – review and editing (supporting). **Nury M Steuerwald:** Conceptualization (supporting); data curation (equal); methodology (equal). **Carol J Farhangfar:** Conceptualization (equal); methodology (equal); resources (equal). **Emily A Baldrige:** Project administration (equal); writing – original draft (equal). **Jennifer H Benbow:** Writing – original draft (equal). **Michael B. Livingston:** Conceptualization (equal); writing – original draft (equal). **Joshua C. Patt:** Supervision (equal); writing – original draft (equal). **William Ahrens:** Data curation (equal); writing – review and editing (equal). **Jeffrey S. Kneisl:** Supervision (equal); writing – original draft (equal). **Edward S Kim:** Conceptualization (equal); investigation (equal).

## FUNDING INFORMATION

Funding was provided by the Paula Takacs Foundation. Funders had no role in study design, data collection, data analysis, data interpretation, or writing of this manuscript.

## CONFLICT OF INTEREST

There are no financial disclosures or conflicts of interest.

## ETHICS STATEMENT

The data collected for this research are unique and truthfully represented. It has been exclusively submitted to this journal and is not being considered for publication elsewhere. This research was conducted with institutional review board (IRB) approval, with informed consent waived due to the retrospective nature of the study. All protected health information for the subjects involved in the study remained confidential.

## Data Availability

The data supporting this study are available upon request from the corresponding author, Megan Jagosky.
